# A missense mutation of ErbB2 produces a novel mouse model of stillbirth associated with a cardiac abnormality but lacking abnormalities of placental structure

**DOI:** 10.1371/journal.pone.0233007

**Published:** 2020-06-03

**Authors:** Heba Shawer, Esther Aiyelaagbe, Christopher Clowes, Samantha C. Lean, Yinhui Lu, Karl E. Kadler, Alan Kerby, Mark R. Dilworth, Kathryn E. Hentges, Alexander E. P. Heazell

**Affiliations:** 1 Division of Evolution & Genomic Sciences, School of Biological Sciences, Faculty of Biology, Medicine and Health, Manchester Academic Health Science Centre, University of Manchester, Manchester, England, United Kingdom; 2 Division of Developmental Biology and Medicine, School of Medical Sciences, Faculty of Biology, Medicine and Health, Manchester Academic Health Science Centre, University of Manchester, Manchester, England, United Kingdom; 3 Wellcome Trust Centre for Cell-Matrix Research, Division of Cell-Matrix Biology and Regenerative Medicine, Faculty of Biology, Medicine, and Health, Manchester Academic Health Science Centre, University of Manchester, Manchester, England, United Kingdom; 4 St. Mary's Hospital, Manchester University NHS Foundation Trust, Manchester Academic Health Science Centre, Manchester, England, United Kingdom; IGBMC/ICS, FRANCE

## Abstract

**Background:**

In humans, stillbirth describes the death of a fetus before birth after 28 weeks gestation, and accounts for approximately 2.6 million deaths worldwide annually. In high-income countries, up to half of stillbirths have an unknown cause and are described as “unexplained stillbirths”; this lack of understanding impairs efforts to prevent stillbirth. There are also few animal models of stillbirth, but those that have been described usually have significant placental abnormalities. This study describes a novel mutant murine model of fetal death with atrial conduction block due to an ErbB2 missense mutation which is not associated with abnormal placental morphology.

**Methods:**

Phenotypic characterisation and histological analysis of the mutant mouse model was conducted. The mRNA distribution of the early cardiomyocyte marker Nkx2-5 was assessed via *in situ* hybridisation. Cardiac structure was quantified and cellular morphology evaluated by electron microscopy. Immunostaining was employed to quantify placental structure and cell characteristics on matched heterozygous and homozygous mutant placental samples.

**Results:**

There were no structural abnormalities observed in hearts of mutant embryos. Comparable Nkx2-5 expression was observed in hearts of mutants and controls, suggesting normal cardiac specification. Additionally, there was no significant difference in the weight, placenta dimensions, giant cell characteristics, labyrinth tissue composition, levels of apoptosis, proliferation or vascularisation between placentas of homozygous mutant mice and controls.

**Conclusion:**

Embryonic lethality in the ErbB2 homozygous mutant mouse cannot be attributed to placental pathology. As such, we conclude the *ErbB2*^*M802R*^ mutant is a model of stillbirth with a non-placental cause of death. The mechanism of the atrial block resulting from ErbB2 mutation and its role in embryonic death is still unclear. Studying this mutant mouse model could identify candidate genes involved in stillbirth associated with structural or functional cardiac defects.

## Introduction

Stillbirth describes the death of a fetus before birth after 28 weeks gestation, and accounts for approximately 2.6 million deaths worldwide annually, occurring at a rate of 18.4 per 1,000 pregnancies in 2015 [1]. Antepartum stillbirth, when fetal death occurs prior to the onset of labour, occurs in 50% of stillbirths in low and middle-income countries and 90% of stillbirths in high-income settings. Despite knowledge of established risk factors such as maternal infection, hypertension, diabetes, maternal obesity and cigarette smoking [2, 3], between 15–60% of stillbirths have an unknown cause, and are referred to as “unexplained” stillbirths [4–7]. Placental abnormalities are the most common abnormality seen in cases of stillbirth with 11–65% of stillbirths being attributed to placental causes [8]. In addition, congenital anomalies are related to 7–9% of stillbirths [3] with an estimated 2–9% of stillbirths associated with structural congenital heart disease (CHD) [9]. CHD is also the most prevalent birth defect that results in infant death and represents one third of fetal congenital anomalies [10–13]. Even in the absence of structural abnormalities, defects in the cardiac conduction system have been reported in cases of sudden infant death and *in utero* fetal death [14–17]. The genetic defects described could cause lethal arrhythmias and sudden cardiac arrest. Despite the association of cardiac conduction defects with infant mortality [18, 19], current knowledge about the contribution of cardiac anomalies and cardiac conduction defects to stillbirth is very limited and the mechanism by which cardiac defects could lead to stillbirth is unclear. Critically, studies have also described associations between the presence of CHD and placental abnormalities [20–22] and many murine models of embryonic lethality have placental defects [23], therefore, developing an animal model of stillbirth without placental abnormalities could improve understanding of physiological and molecular mechanisms of fetal death specifically relating to the role of cardiac defects.

We have previously described a mutant mouse model, *l11Jus8*, with atrial conduction defects that resulted in embryonic death at embryonic day (E) 12.5–13 [24]. However, the contribution of cardiac dysfunction to the observed late embryonic death and the underlying molecular mechanisms is still unclear. This mutant mouse model is homozygous for Met-802-Arg missense mutation in the Mus musculus v-erb-B2 erythroblastic leukemia viral oncogene homolog-2 (ErbB2) gene [24]. It is hypothesised that the embryonic death of *ErbB2*^*M802R*^ mutants at late gestation resulted from similar atrial conduction defects to those observed in the *l11Jus8* mouse. This study aimed to investigate mechanisms underlying embryonic death in this mouse model; specifically, i) whether there are abnormalities of cardiac structure and ii) whether there are placental abnormalities as these are the most frequent cause of human stillbirth. We have shown that placental developmental is not impeded in the *ErbB2*^*M802R*^ mutant mouse, suggesting that it is a suitable model for studying non-placental pregnancy loss. Since animal models of stillbirth not attributed to altered placental structure and function are rare, the *ErbB2*^*M802R*^ mutant mouse shows promise for providing a new system in which to investigate stillbirths related to cardiac abnormalities.

## Materials and methods

### Ethics statement

Experiments using animals were performed in accordance with legislation in the UK Animals (Scientific Procedures) Act of 1986 (PPL 70/8858 to Graham Morrissey). Experiments were approved by the University of Manchester Animal Welfare and Ethical Review Body.

### Mice

The *l11Jus8 and ErbB2*^*M802R*^ mouse line was maintained in the Biological Service Facility of the University of Manchester, UK, with local ethical approval and according to UK Home Office requirements (Home Office project licence 70/8858). The *l11Jus8* mouse strain was generated by random ENU mutagenesis [25]. The *ErbB2*^*M802R*^ mouse line was maintained on a mixed C57BL/6 and 129S5/SvEvBrd background as described previously [24]. The *ErbB2*^*M802R*^ mutation was maintained in trans to a balancer chromosome and thus wild type embryos are not obtained from the crosses performed [25], as embryos lacking the *ErbB2*^*M802R*^ mutation are homozygous for the embryonic lethal balancer mutation in *Wnt3*. Homozygous balancer embryos were excluded from this study. Approximately 45 dams were used for the study. Embryo and placental weights were measured immediately after dissection at E12.5. Yolk sacs were used for genotyping using Sanger sequencing to confirm the Met-802-Arg missense mutation in the *ErbB2* gene as previously described [24]. The *ErbB2* Primers used for sequencing were *ErbB2*-Forward: 5’-TCCCTCTGTTCCCTTGTCTG-3’, and *ErbB2*-Reverse: 5’-AACCCCCAAAGCACATACCT-3’.

### Histology and immunohistochemical staining

Embryos were formalin-fixed, paraffin-embedded and cut into 5μm-thick sections. Standard histological techniques for Harris’ haematoxylin and eosin Y (1%) (Sigma-Aldrich Co.) (H&E) staining were performed. Sections were imaged on a Zeiss Axioplan2 upright microscope. Images were acquired using an Axiocam MrC camera (Zeiss) through Axiovision v4.8.2 software (Zeiss), and processed using Fiji ImageJ software (http://imagej.net/Fiji/Downloads). Cardiac area measurements were made from 5x magnification images of E11.5 ventricular myocardium, atrioventricular canal (AVC) and atrial myocardium stained with nuclear fast red. Images were acquired using Image-Pro Insight software (Media Cybernetics Inc., Version 9), from homozygous mutants (n = 5) and heterozygous controls (n = 9). Cardiac area measurements were performed using ImageJ software (https://www.imagej.nih.gov/ij). At least 3 sections in each cardiac area were measured for each embryo analysed and mean area was obtained. Ventricular myocardium, AVC and atrial myocardium tissue area were measured in heterozygote (n = 9) and homozygous mutant (n = 5) embryos at E11.5.

For placental analysis of heterozygous and homozygous mutants, formalin-fixed paraffin-embedded placental tissue was cut in the sagittal plane into 5μm-thick tissue sections. For immunohistochemical staining, tissue sections were deparaffinised and rehydrated, and antigen retrieval was achieved by heating in sodium citrate buffer (pH 6). Sections were incubated in 3% hydrogen peroxide (VWR International), washed and then blocked using non-immune block (88% TBS-Tween (0.1%), 10% swine serum and 2% human serum). Antibodies against Ki67 (1:100, Abcam ab15580), Active caspase 3 (1:500, Cell Signalling Technology 9664), CD34 (1:200, Abcam ab81289), monocarboxylate transporter 4 (MCT4, 1:200, Millipore AB3316P) and Griffonia Simplicifolia Lectin I (GSL I) isolectin B4 (IL-B4, 1:200, Vector) were incubated at room temperature for 1h or overnight at 4°C. Sections were washed and then incubated in biotinylated polyclonal swine anti-rabbit secondary antibody (1:200, Dako) for 30 minutes. After incubating in avidin peroxidase, the tissue sections were washed, and visualised using Diaminobenzidine (DAB) (Sigma-Aldrich Co.). Sections were counterstained using standard protocols with either haematoxylin alone or in combination with eosin (H&E). All images were analysed using HistoQuest (Version 3.5.3.0185, Tissue Gnostics) or Qupath (v0.1.2) software for unbiased quantification [26]. The total placental area measured was the sum of the labyrinth and junctional zones. This was performed by measuring the MCT4^+^ area (labyrinth zone) and using the IL-B4 stained image to confirm the junctional-decidua interface. The labyrinth and junctional zone thickness were averaged from measurements at the start, end, and at 500μm intervals along the regions, then expressed as relative to the placenta area (S1A and S1B Fig). Spongiotrophblast inclusions in the labyrinth were identified from IL-B4 and haematoxylin morphology. The total area of spongiotrophoblast inclusions was calculated and then expressed relative to the labyrinth area (S1A and S1C Fig). The labyrinth basal membrane was identified by IL-B4 staining and was quantified in 5x areas of 15,625μm^2^ with a DAB^+^ area detection protocol (S1A and S1D Fig). Using the same sampling method cell density in the labyrinth was calculated from a haematoxylin cell detection protocol (S1A and S1E Fig). Giant cells in the junctional zone were identified by morphology and quantified by measuring the longest dimension of each nuclei (S1A and S1G Fig). The total number of giant cells was expressed relative to the placenta area. Quantification of Ki67 and Cas3 staining was achieved by quantifying the total nuclei (haematoxylin) and the number of cells positive for the primary antibody (DAB). The number of nuclei positively stained by primary antibody are presented as a proportion of total nuclei. Quantification of CD34 was achieved by quantifying the area of DAB^+^ tissue and the total labyrinth zone area. The proportion of CD34^+^ area in the labyrinth zone indicates the proportion of endothelial cells and hence the relative fetal vascularisation in the placentas.

### Electron microscopy

Embryonic hearts from of heterozygous (n = 3) and homozygous mutant (n = 4) at E11.5 were fixed in 2% glutaraldehyde in PBS (pH 7.0) then stored at 4°C until further processing, embedding, sectioning and imaging as previously described [27]. Sections were examined using Tecnai 12 Biotwin transmission electron microscope (FEI UK Limited, Cambridge), and images were acquired using an Orius SC1000 camera (11 Megapixels, 4008 x 2672, Gatan, Abingdon).

### In situ hybridisation

Whole mount *in situ* hybridisation was performed as previously described [28], to assess the localisation of Nkx2-5 at E10.5 in heterozygous (n = 3) and *l11Jus8* homozygous mutant embryos (n = 3).

### Quantitative real-time reverse transcription (RT)-PCR

Murine cardiac tissues were pooled into four groups (atria from homozygous mice (n = 3), atria from heterozygous mice (n = 3), ventricles from homozygous mice (n = 3) and ventricles from heterozygotes (n = 3)). Atria and ventricles were harvested from homozygous and heterozygous mutant mice, placed in RNA*later* stabilisation solution directly after being dissected and then stored at -20°C. Total RNA was extracted using mirVana miRNA Isolation Kit (Life technologies), and genomic DNA was digested using Turbo DNA-free kit (Life technologies), according to manufacturers’ protocol. The quantity and quality of the extracted RNA was assessed by Nanodrop (Thermo Scientific) as previously described [29]. The total RNA was reverse transcribed using AffinityScript cDNA Synthesis Kit (Agilent Technologies). Quantitative PCR was performed to assess the steady-state levels of mRNAs encoding for six cardiac ion channels (Scn5a, Kcnh1, Kcnq1, Hcn4, Cacna1g, and Kcnk3). The house-keeping gene TATA-Box Binding Protein (TBP) was used as an endogenous control. QPCR was performed in three separate experimental repeats for each pooled sample in each group, using Brilliant III Ultra-Fast SYBR Green QPCR Master Mix (Agilent technologies) or GoTaq qPCR master mix (Promega). Primer sequences used were Scn5a-Forward (F): 5’-GGAGTACGCCGACAAGATGT-3’, Scn5a-reverse (R): 5’-ATCTCGGCAAAGCCTAAGGT-3’, Hcn4-F: 5’-GCTTTCCCGCCTCATTCGAT-3’, Hcn4-R: 5’-CCCCAGGAGTTATTCACCATGC-3’, Kcnq1-F: 5’-ACCACTTCACCGTCTTCCTCA-3’, Kcnq1-R: 5’-CCAGAGGCGGACCACATATT-3’, Cacna1g-F: 5’-GAACGTGAGGCCAAGAGT-3’, Cacna1g-R: 5’-GCTCGTAAGCGTTCCCCT-3’, Kcnk3-F: 5'- CGGCTTCCGCAACGTCTAT-3', Kcnk3-R: 5'- TTGTACCAGAGGCACGAGCA-3', Kcnh1-F: 5'-TTTCTGGAGAACATCGTGCGG-3', Kcnk3-R: 5'-CTCCATACATAAAACTGCAGGCG-3', Tbp-F: 5’-CACAGGAGCCAAGAGTGAAGA-3’, and Tbp-R: 5’-CACAAGGCCTTCCAGCCTTA-3’.

### Statistical analysis

Statistical analyses were performed using GraphPad Prism 7 statistical packages (GraphPad Software Inc.). For statistical comparisons, Mann–Whitney U test or Kruskal-Wallis (with Dunn’s post-hoc test) was performed to analyse the difference between two or multiple groups respectively as appropriate, with statistical significance considered at p<0.05. The relative expression of cardiac ion channels was calculated using comparative ΔΔCT method [30] and are presented as log transformed log10 (2^-ΔΔCT^) values. Data from Caspase 3 immunostaining is presented on logarithmic scale.

## Results

### Morphological features of mutant embryos

**Fig 1:**
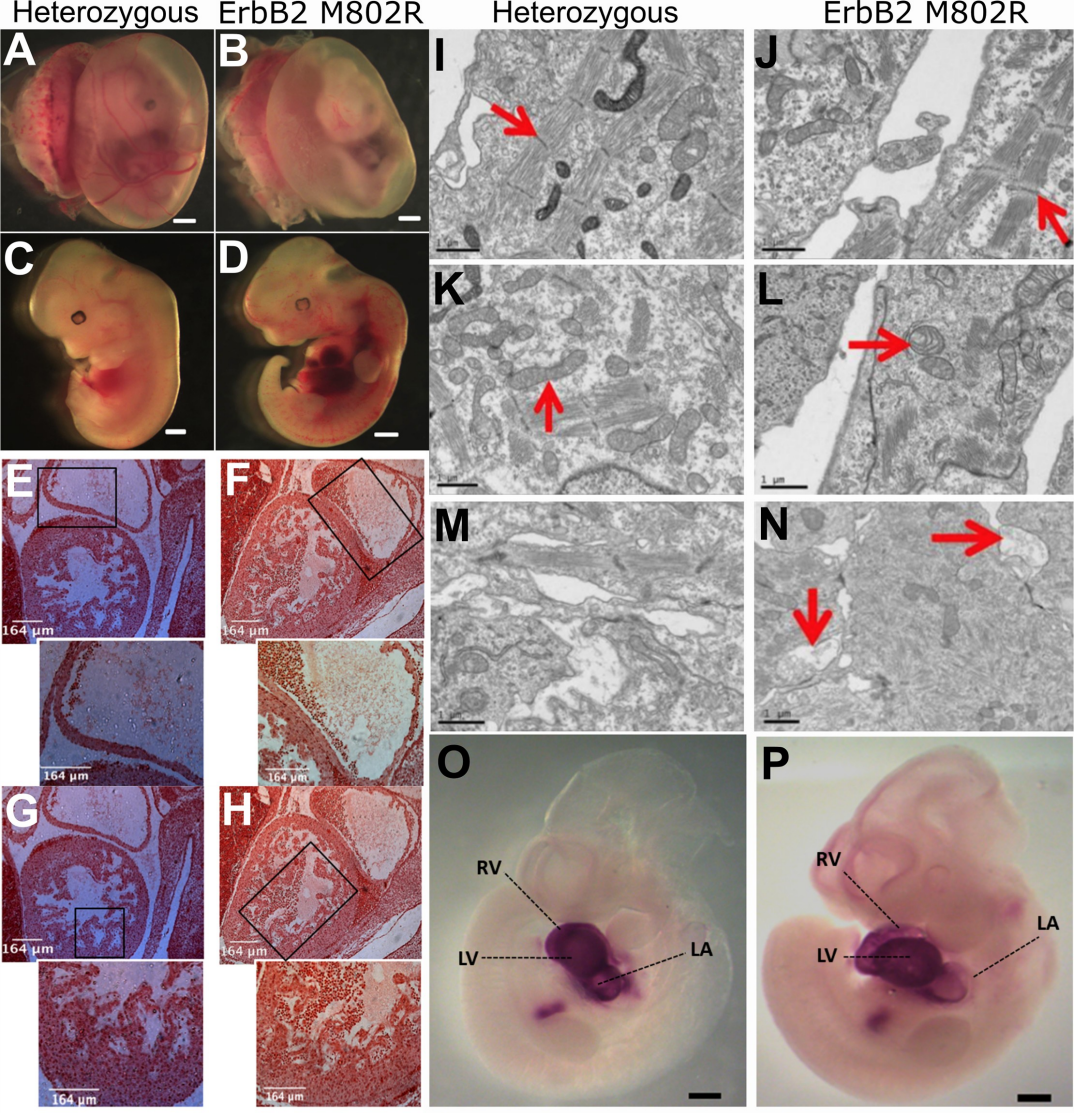
Morphological and ultrastructural and molecular analysis. **(A)** Heterozygous and **(B)** Homozygous *ErbB2*^*M802R*^ mutant embryo yolk sacs showing a difference in vascularisation. **(C)** Heterozygous and **(D)** *ErbB2*^*M802R*^ homozygous mutant embryos at E12.5 showing haemorrhage in the thoracic cavity in Scale bar **(A-D**) = 1 mm. **(E-H)** Haematoxylin and Eosin stained sagittal sections of E12.5 hearts show no structural abnormalities in the ventricles from **(E)** heterozygous, and **(F)** homozygous *l11Jus8* mutants, or the atria from **(G)** heterozygous and **(H)** homozygous *l11Jus8* mutants. **(E-H)** Scale bar = 164 μm. **(I-N)** Representative ultrastructural features at E11.5 observed in mutant atrial myocardium. **(I,J)** Images of intact repeating units of sarcomeres (red arrows) in **(I)** heterozygous and **(J)** homozygous mutant embryos. **(K,L)** Swollen mitochondria in homozygous mutants (red arrows). **(M,N)** Presence of electron lucent membrane bound vesicles in homozygous mutants in between neighbouring myocardium cells or being engulfed by or excreted by myocardium cells (red arrows). **(I-N)** Scale bars = 1μm. **(O-P)** Nkx2-5 *in situ* hybridisations in E10.heterozygous and homozygous *l11Jus8* mutants. LA—Left Atrium, LV—Left Ventricle, RA—Right Atrium, RV—Right Ventricle. Scale bar = 1mm.

Phenotype and sequence screening analysis was previously performed to characterize the *l11Jus8* mutant model [24]. The homozygous *l11Jus8* mutant embryos exhibited haemorrhage in the thoracic cavity, as well as subcutaneous haemorrhage throughout the body. Around 36% of homozygous *l11Jus8* mutants displayed thoracic haemorrhage and absence of yolk sac vascularisation at E12.5 [24]. Here, we show that homozygous *ErbB2*^*M802R*^ mutants also displayed haemorrhages in the thoracic cavity and defects in yolk sac vessels (Fig 1A–1D) in agreement with the previously described *l11Jus8* mutant phenotype [24]. The overall ratio of survived heterozygous: homozygous *ErbB2*^*M802R*^ mutants varied from 5:1 to 1:2. There was no difference between the fetal weights of heterozygous and homozygous mutants (Heterozygous median weight 66.7g Interquartile range (IQR) 54.2g - 78.6g and homozygous mutants median 69.4g IQR 61.1g - 72.8g; p = 0.88). Histological examination of E12.5 hearts from heterozygous (n = 3) and *l11Jus8* homozygous mutant (n = 3) showed no structural abnormalities (Fig 1E–1H).

**Fig 2:**
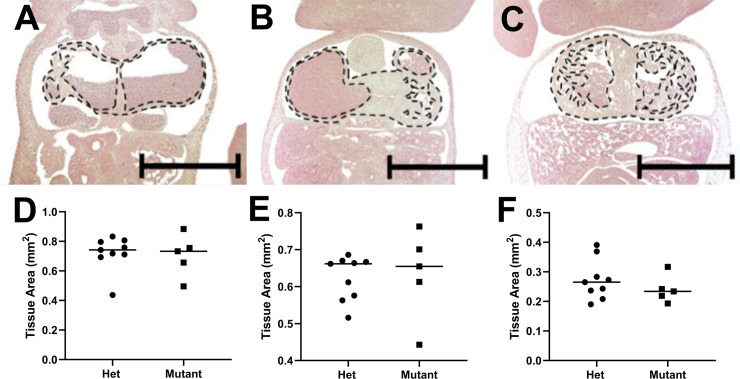
Morphological features of the heterozygous controls and homozygous mutants. Representative image of coronal sections and graphical representation of **(A,D)** atrial myocardium area, **(B,E)** atrioventricular canal tissue area, and **(C,F)** ventricular myocardium at E11.5 for wild type (blue bars), heterozygote (red bars) and *l11Jus8* (L8) mutant embryos (green bars). (heterozygote n = 9 and homozygous mutant n = 5) **(A-C)** Scale bars = 1mm. Images for each genotype were taken at equivalent depth through the heart. Dotted lines indicate area measured.

Cardiac tissue areas were assessed in homozygous *l11Jus8* mutants (n = 5) and heterozygous controls (n = 9) at E11.5 to investigate the possibility of myocardial hypoplasia in mutants; no significant differences were observed in ventricular myocardium, atrioventricular canal (AVC) or atrial myocardium tissue areas between the groups (Fig 2). Possible intracellular defects and alterations to the structural integrity of sarcomeres were assessed by electron microscopy of heterozygous (n = 3) and homozygous *l11Jus8* mutant (n = 4) E11.5 atrial myocardium. Intact sarcomeres were observed in both groups encompassing variable numbers of repeating units bordered at each end by an electron dense Z-disc with two electron lucent I-bands at either side of an A-band with an electron lucent M-line at their centre (Fig 1I and 1J). Similarly, the intracellular organelles and components showed similar morphology in both genotypes. Nonetheless, in two homozygous *l11Jus8* mutants, the mitochondria appeared swollen and occupied approximately double the volume of the heterozygous controls whilst retaining the same number of cristae which made them appear sparse in comparison (Fig 1K and 1L). Moreover, several multi-vesicular bodies (~2μm in length and width) were frequently observed in intercellular spaces between neighbouring myocardial cells in three homozygous mutants and not in any of the heterozygous controls (Fig 1M and 1N). These multi-vesicular bodies are consistent with descriptions of microparticles [31].

To determine if defects in the differentiation of cardiomyocytes could be contributing to the observed cardiac electrical defects in *l11Jus8* mutants, the mRNA distribution of the core cardiogenic transcription factor and early cardiomyocyte marker *Nkx2-5* (NK2 Transcription Factor Related, Locus 5) was assessed by *in situ* hybridisation in heterozygous (n = 3) and *l11Jus8* homozygous mutant (n = 3) embryos at E10.5. Nkx2-5 expression was uniformly present throughout the developing heart in left and right ventricular, atrial chamber myocardium and outflow tract (OFT) myocardium (Fig 1O and 1P). The expression pattern in mutants was indistinguishable from the heterozygous controls. These results imply normal cardiac specification and maintained involvement of Nkx2-5 in cardiac myocardium gene expression.

Since *ErbB2*^*M802R*^ homozygous mutants had a similar phenotype to the *l11Jus8* mutant model which displayed atrial conduction block [24], but did not show major defects in cardiac structural morphology, we conducted a preliminary analysis of mRNA expression levels of cardiac ion channels. There was no statistically significant difference in expression levels of any of mRNA for *Scn5a*, *Hcn4*, *Kcnh1*, *Kcnq1*, *Kcnk3*, *or Cacna1g* between the atria of the homozygous mutant mice and the atria of heterozygotes or between their ventricles (S2 Fig).

### Absence of features of abnormal placental morphology in ErbB2^M802R^ mutant embryos

**Fig 3:**
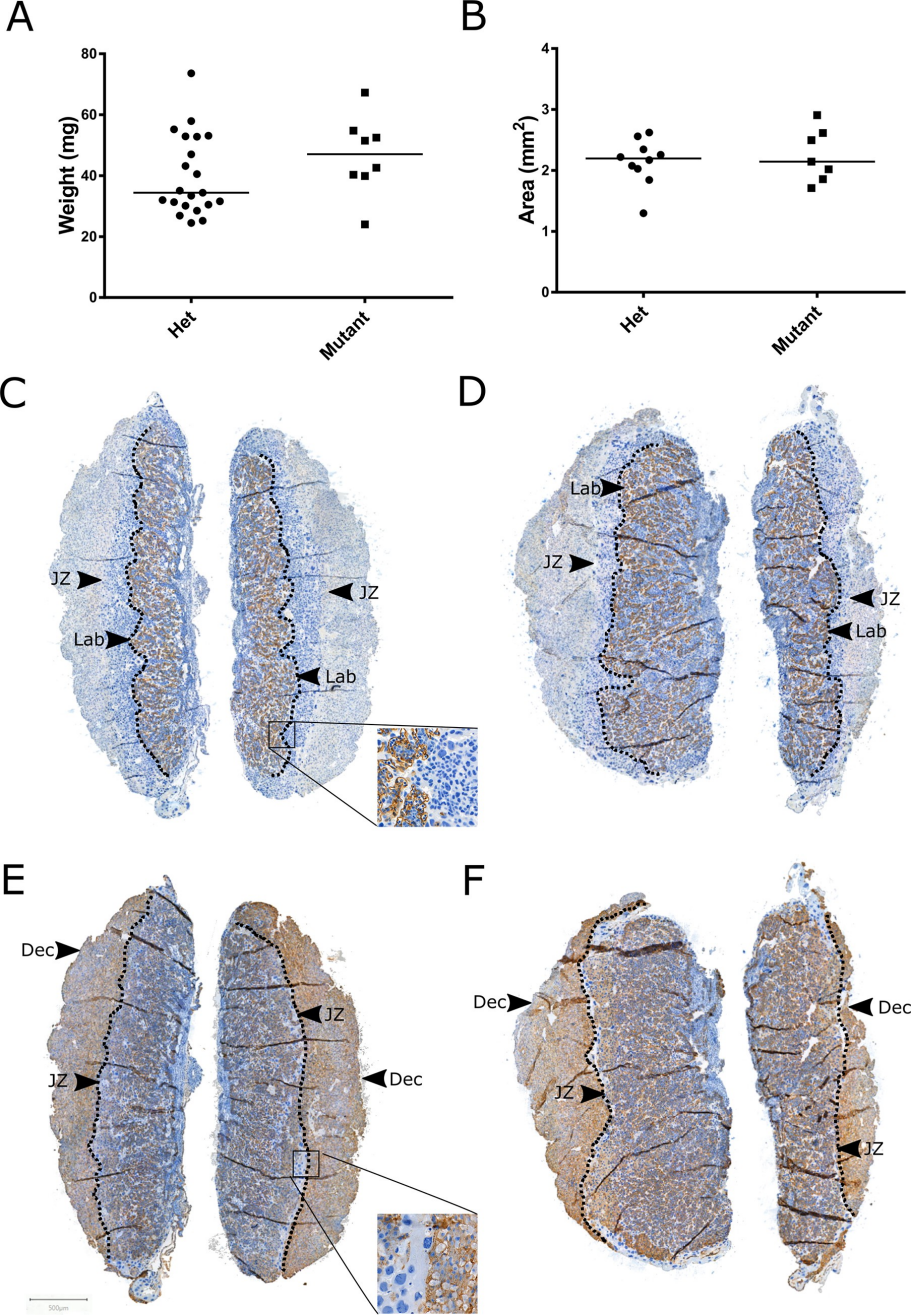
Placenta weight and tissue area in heterozygous and homozygous *ErbB2*^*M802R*^ mutants at E12.5. Quantification of **(A)** placental weight **(B)** placental area. Data expressed as median with data points. Example **i**mages of **(C,E)** heterozygous and **(D,F)** homozygous mutant mouse placentas immunostained with **(C,D)** MCT4 and **(E,F)** IL-B4 and captured at x1 magnification. Arrows indicate the transitions at the junctional zone **(JZ)**, labyrinth **(Lab)**, and decidua **(Dec)**. Scale bar = 500μm.

**Fig 4:**
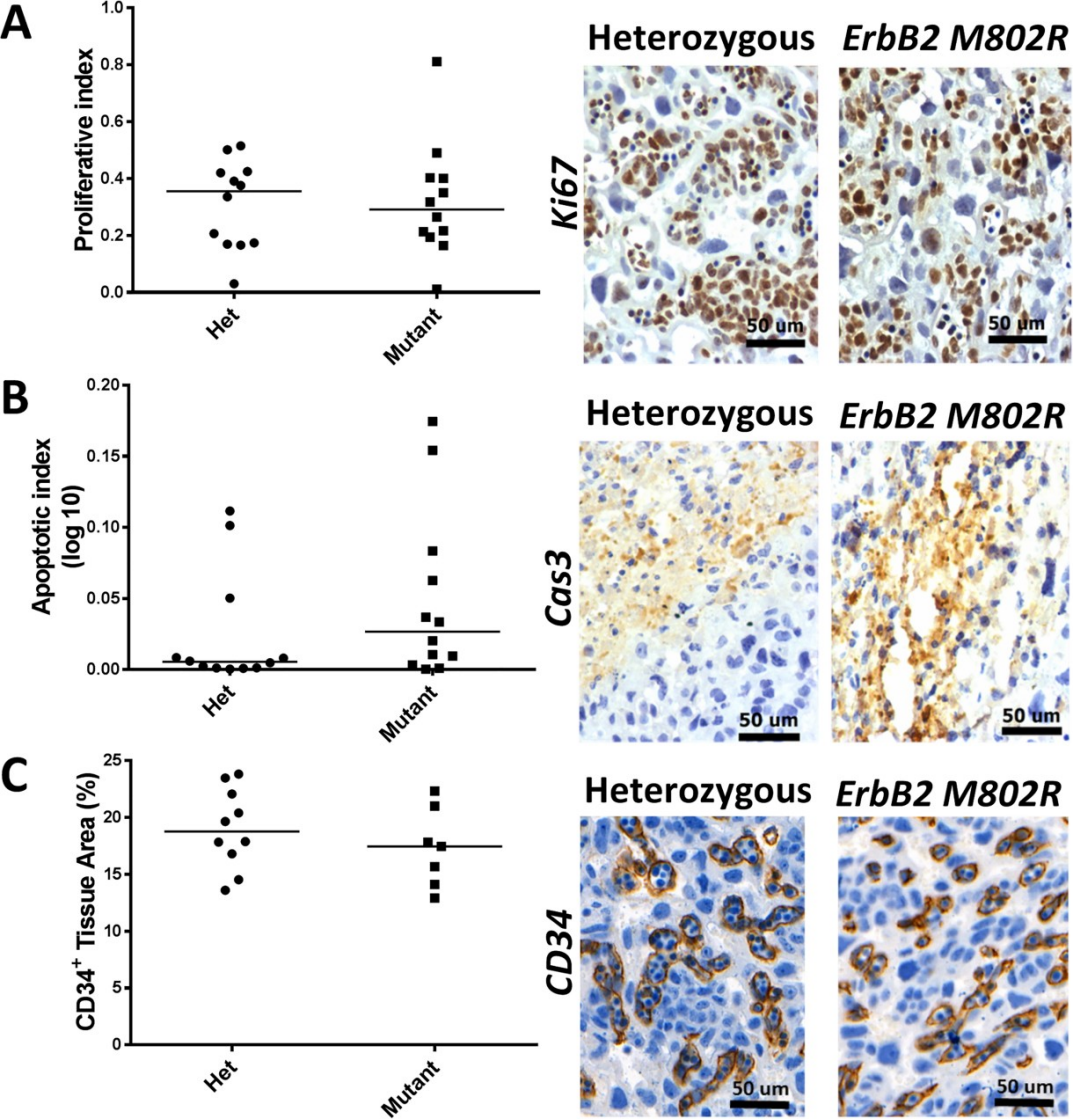
Placenta phenotype in heterozygous and homozygous *ErbB2*^*M802R*^ mutants at E12.5. Quantification of **(A)** proliferative index (Ki67)**, (B)** apoptotic index (active Caspase 3), **(C)** vascularisation index (CD34) in placentas from heterozygous (Het) and homozygous (Mutant) embryos. Scale bar 50 μm. **A** and **B** are expressed as ratio of stain +ve nuclei/total nuclei, **E** is expressed as the percentage of CD34^+^ tissue in the labyrinth zone. Data expressed as median with data points.

As placental dysfunction is a major cause of *in utero* fetal death, the placentas of *ErbB2*^*M802R*^ mutants were examined for alterations in various aspects of placental structure and turnover. The morphology of the placental zones in mice often differs between pregnancies with normal and pathological outcomes [32, 33]. Here, placental weights were not significantly different between heterozygous (n = 21) and homozygous mutants (n = 8; Fig 3A). Tissue area of the junctional and labyrinth zones was assessed by MCT4 (Fig 3C and 3D) and IL-B4 (Fig 3E and 3F) staining. There was no significant difference in the total placental area (Fig 3B), the thickness of the labyrinth and junctional zone, or the labyrinth:junctional zone ratio between heterozygous (n = 11) and homozygous mutants (n = 10) (Fig 3, Table 1, S1 Fig). There was also no difference in spongiotrophoblast inclusions, labyrinth basal membrane area, and labyrinth cell density (Table 1, S1 Fig). The frequency and nuclei size of giant cells in the junctional zone was also unchanged (Table 1, S1 Fig). In cases of human late fetal death, especially those secondary to placental fetal growth restriction reduced proliferation and vascularisation is seen in the villi [34]. Consequently, these features were assessed in the mouse model; there were no differences in proliferation (Fig 4A, S3 Fig), or apoptosis (Fig 4B, S4 Fig), expressed as ratio of positive nuclei to the total number of nuclei, between heterozygous and homozygous mutants (n = 12/group). Furthermore, no differences were seen between placentas assessed by immunostaining CD34 as a marker of blood vessels. In the labyrinth there was no significant difference in the proportion of CD34^+^ tissue (Fig 4C, S5 Fig) between heterozygous (n = 10) and homozygous mutants (n = 7).

**Table 1:** Investigation of fetal and placental parameters in heterozygous (Het) and homozygous (Mutant) ErbB2 mutant mice.

Parameter	Unit	Het	Mutant	*P*
Labyrinth Area	mm^2^	1.33	1.33	0.56
Labyrinth Thickness	Thickness/Placenta Area (μm)	684	557	0.20
Junctional Zone Area	mm^2^	0.89	1.09	0.65
Junctional Zone Thickness	Thickness/Placenta Area (μm)	249	233	1.00
Labyrinth:Junctional Zone Ratio	Ratio	1.9	1.5	0.28
Spongiotrophoblast Inclusion Area	Percentage of Labyrinth (%)	3.1	2.7	0.92
Labyrinth Basal Membrane Area	IL-B4^+^ Area	29.0	32.2	0.31
Labyrinth Cell Density	Cells/mm^2^	8,832	8,819	0.79
Giant Cell Size	Nuclei Size (μm)	23.8	24.9	0.97
Giant Cells	Frequency/Placenta Area	83.9	70.7	0.76

Analysis included 11 Het and 10 Mutant mice. Where stated data are presented in proportion to the placenta area. Data are median values and *P* values are the result of Mann-Whitney testing.

## Discussion

Although various pathologies are associated with stillbirth, including placental abnormalities, maternal infection and congenital anomalies, approximately 15 to 60% of stillbirths still have an unknown cause [4–7]. Cardiac origins of fetal death are a logical avenue to explore as structural CHD represents one third of fetal congenital anomalies and they are associated with stillbirth [10–13]. Furthermore, defects in the cardiac conduction system have been reported in cases of sudden infant death and *in utero* fetal death in the absence of structural cardiac abnormalities [14–17]. Despite the implication of genetic cardiac conduction defects in infant mortality [18, 19, 35], current knowledge about the contribution of structural or functional cardiac defects to stillbirth, and the mechanism by which such defects could lead to stillbirth, is unclear.

The absence of suitable animal models of otherwise unexplained stillbirth, particularly those with no evidence of placental dysfunction contributes to the poorly understood aetiology of this pregnancy outcome. Perez-Garcia et al. evaluated 103 different murine models and found that 68% of those that were lethal at or after mid-gestation had abnormal placental morphology; there was also a strong relationship between abnormal brain, heart and vascular development and placental defects [23]. Therefore, developing an animal model of non-placental stillbirth could improve understanding of physiological and molecular mechanisms underlying stillbirth associated with cardiac anomalies. Recently, murine models of pregnancy that resemble certain risk factors for stillbirth, for instance infection [36–38], prolonged post-term pregnancy [39], advanced maternal age [40] and maternal psychological stress [41] have been developed to investigate the underlying mechanisms and aetiologies to fetal death *in utero*. Although CHD is related to stillbirth, there have been, until now, no animal models of stillbirth that represent cardiac defects without significant associated placental defects [32].

The *l11Jus8* mutant mouse model has atrial conduction block that resulted in embryonic lethality at late gestation (E12.5–13) [24]. Using this murine model, the underlying causes of the atrial conduction defects and their potential contribution to the late embryonic death was investigated. The *l11Jus8* mutant strain is homozygous for a hypomorphic M802R mutation in the ErbB2 gene [24]. The missense mutation in this mouse model is located in the Serine-Threonine/Tyrosine kinase domain of the ErbB2 gene, within a region showing high conservation among multiple species, including mouse and human. Therefore, findings in the mouse model could be relevant to human disease. ErbB2 is involved in signalling cascades that regulate various essential processes including cardiac conduction system development [42], ventricular myocyte differentiation [43], and calcium uptake in the sarcoplasmic reticulum [44]. Nonetheless, the role of ErbB2 in the development of the atrial conduction system and the molecular basis of atrial conduction block in the mutant mouse model remain elusive. Homozygous *ErbB2*^*M802R*^ mutants, and not heterozygous littermates, displayed haemorrhages in the thoracic cavity and defects in yolk sac vessels. These observations are in agreement with the previously described *l11Jus8* mutant phenotype [24]. However, unlike the previously reported homozygous ErbB2 knockout mutants, the *ErbB2*^*M802R*^ homozygous mutants did not show any cardiac structural defects. Prior studies show both the Erbb2 null mutants and kinase-dead ErbB2 homozygous mutant mice have defects in cardiac trabeculation [45, 46]. However, cardiac trabeculae were clearly observed in histological sections of both heterozygous and homozygous *l11Jus8* mutants. Differentiation of cardiomyocytes was studied in *l11Jus8* mutant embryos via examining the mRNA distribution of the early cardiomyocyte marker Nkx2-5 and did not show different expression pattern among the different genotypes, suggesting normal cardiac specification and implies maintained involvement of Nkx2-5 in cardiac myocardium gene expression regulatory loops. However, further studies are needed to analyse the expression of more mature cardiomyocyte differentiation genes to elucidate the nature of cardiomyocyte differentiation in this mutant mouse model.

Ultrastructural analysis of the intracellular organelles in hearts of *l11Jus8* mutants using electron microscopy showed swollen mitochondria in half of the examined *l11Jus8* mutants relative to heterozygous controls. Emerging evidence supports the association of mitochondrial structural and functional abnormalities with cardiac diseases including cardiac conduction dysfunction, and cardiomyopathy [47, 48]. This association could be attributed to the high energy demand of the cardiac muscles. Therefore, defects of the mitochondrial function increase the risk of developing cardiac diseases [49, 50]. Further analysis is still needed to investigate the involvement of the mitochondrial structural difference between *l11Jus8* mutants and heterozygous controls in the development of cardiac conduction defects in *l11Jus8* mutants.

There is a heart-placenta axis early in pregnancy, which suggests that both organs grow synergistically [51] and their development pathways share common genes and molecules [51, 52]. Thus, abnormal placental development may lead to abnormal cardiac development or vice versa as the volume of placental blood flow is related to cardiac output and thus fetal growth [51, 53, 54]. Increased resistance in placental vasculature causes remodelling of the developing heart which may lead to congenital heart disease [53]. Knowing that placental dysfunction is the most frequent cause of human stillbirth [8] and due to the multifactorial aetiology of stillbirth, placental abnormalities in the *ErbB2*^*M802R*^ mouse model were investigated. Critically, none of the key features of placental morphology investigated in these studies, including placental weight, placenta dimensions, giant cell characteristics, labyrinth composition, cell turnover, apoptosis and vascularisation, were found to be altered in homozygous mutant relative to heterozygous embryos, suggesting that embryonic death was not attributed to alterations in placental structure in *ErbB2*^*M802R*^ mutants. Many models of embryonic lethality caused by non-placental pathology do not include placental characterisation, which makes comparison of placental findings between murine models of stillbirth difficult. However, our findings clearly differ from descriptions of fetal death in mouse models which are associated with placental dysfunction, which display altered placental structure, increased apoptosis and dysplasia of spongiotrophoblast and giant cells and altered amino-acid transport [32, 33, 40]. While we have found evidence that the morphology of the placenta including the vasculature is not significantly altered between heterozygous and homozygous mutants, it is possible that the cardiac defect could cause insufficient blood flow in the fetal vessels of the placenta. Further studies using blood pressure measurements in the mother and Doppler ultrasound of the fetal vessels may indicate if this is a factor in the stillbirth phenotype.

Mice share many features with humans including their placental development [55]. Thus, murine models are widely used in studying adverse pregnancy outcome and cardiovascular disorders. Nonetheless, human development and physiology differs from that of the other mammals thus the findings of this study should be interpreted with caution. For instance, the mouse heart beats ten times faster than that of human [56], which implies differences in the cardiac electrophysiological features. The repolarisation phase in mouse heart is maintained by transient outward currents, unlike the repolarisation in human heart that is generated by delayed rectifier currents [57, 58]. Therefore, findings regarding cardiomyocyte repolarisation in murine models could not be extrapolated in humans. Moreover, due to the short gestation period in mice, the late embryonic lethality observed at E12.5–13 does not precisely reflect late fetal death in human stillbirths. Nonetheless, it could possibly model human fetal death at earlier gestation (e.g. second trimester). Despite the difference between human and mouse physiology, the genetic manipulation possible in murine studies provided valuable information about pregnancy-related pathologies, that otherwise could have been unobtainable. The mouse model described in the current study is novel, as it resembles many features of human stillbirths. Tenin *et al*. revealed that the atrial conduction block in mutant embryos underlies the cardiac dysfunction and embryonic death [24]. The preliminary analysis of cardiac ion channels found no altered expression levels of the examined cardiac ion channels in mutant hearts, which establish the basis for future research to study the underlying molecular mechanisms that resulted in atria conduction defects in this model, and to investigate the role of ErbB2 in the development of the atrial conduction system. Therefore, further studies need to be conducted with relatively larger sample size and molecular analysis to elucidate the cellular cardiac and placental mechanisms underlying embryonic lethality. These could then be expanded to cases of unexplained stillbirth in humans to explore cardiac ion channelopathies.

In conclusion, the present study describes a murine model that shares many characteristics with late stillbirth in humans to investigate the contribution of cardiac dysfunction to otherwise unexplained fetal death. The mutant embryos displayed cardiac haemorrhage and defects in yolk sac vascularisation. There were no significant differences observed in the expression level of the cardiac ion channels examined in *ErbB2*^*M802R*^ mutant hearts. Therefore, the mechanism of the atrial block resulting from ErbB2 mutation is still unclear. The absence of morphological placental defects in the *ErbB2*^*M802R*^ mutant mouse render it an interesting animal model of stillbirth of non-placental causes. Studying this mutant mouse model holds promise to identify candidate genes involved in stillbirth associated with cardiac defects, if candidate genes are identified these could be examined in human hearts in cases of unexplained stillbirth. Further studies are still needed to promote our understanding of the underlying causes of stillbirth in human fetuses both with, and without cardiac anomalies.

## Supporting information

S1 Fig Methodology for quantitative phenotyping of mouse placentas in heterozygous and homozygous mutant mice at E12.5. Labyrinth **(Lab)**, junctional zone **(JZ)**, and decidua **(Dec)** placenta areas are indicated. Annotations throughout indicate junctional zone area **(green)**, junctional zone thickness **(pink lines)** labyrinth area **(black)**, labyrinth thickness **(blue lines)** spongiotrophoblast inclusions **(yellow)**, giant cell nuclei **(straight red lines)**, labyrinth quantification areas **(turquoise squares)**. **(A)** Whole mouse placenta section stained with IL-B4 and H&E at 1x magnification. **(B)** Haematoxylin channel at 5x magnification showing junctional zone **(pink lines)** and labyrinth **(blue lines)** thickness measurements and contrasting nuclei morphology in the placenta areas. **(C)** IL-B4 and H&E channels at 10x magnification showing a spongiotrophoblast inclusion **(yellow)**. **(D)** DAB channel at 10x magnification showing labyrinth basal membrane quantification **(red enclosures)**. **(E)** Haematoxylin channel at 10x magnification showing labyrinth cell quantification (red circles). **(F)** DAB channel at 5x magnification showing contrasting IL-B4^+^ tissue morphology in the placenta areas. **(G)** IL-B4 and H&E channels at 10x magnification showing giant cells **(straight red lines)**.
(TIF)

S2 Fig Quantitative real-time PCR analysis of cardiac ion channels mRNA levels in heterozygous (Het) and homozygous *ErbB2*^*M802R*^ mutant hearts at E12.5. Relative mRNA expression of **(A)** Scn5a **(A)**, **(B)** Hcn4 **(B)**, **(C)** Kcnh1 **(C)**, **(D)** Kcnq1 **(D)**, **(E)** Kcnk3 **(E)**, or **(F)** Cacna1g **(F)** in heterozygous and mutant hearts presented as log transformed values log10 (2^-ΔΔCT^). (Het—Heterozygous, V—Ventricles).
(TIF)

S3 Fig Ki67 staining in mouse placentas in heterozygous and homozygous mutant mice at E12.5. Example images of the tissue sections of **(A)** heterozygous and **(B)** homozygous mutant mouse placentas captured at x1 magnification. Arrows indicate Ki67 positive cells. Scale bar = 500μm.
(TIF)

S4 Fig Cas3 staining in mouse placentas of heterozygous and homozygous mutant mice at E12.5. Example images of the tissue sections of **(A)** heterozygous and **(B)** homozygous mutant mouse placentas captured at x1 magnification. Arrows indicate Cas3 positive cells. Scale bar = 500μm.
(TIF)

S5 Fig CD34 staining in mouse placentas of heterozygous and homozygous mutant mice at E12.5. Example images of the tissue sections of **(A)** heterozygous and **(B)** homozygous mutant mouse placentas captured at x1 magnification. Arrows indicate vessels. Scale bar = 500μm.
(TIF)

S1 Data (XLSX)
