# Supplementary figures and images for: A missense mutation of ErbB2 produces a novel mouse model of stillbirth associated with a cardiac abnormality but lacking abnormalities of placental structure
Source: PLoS One. 2020 Jun 3;15(6):e0233007. doi: 10.1371/journal.pone.0233007 (PMC7269201; doi:10.1371/journal.pone.0233007)

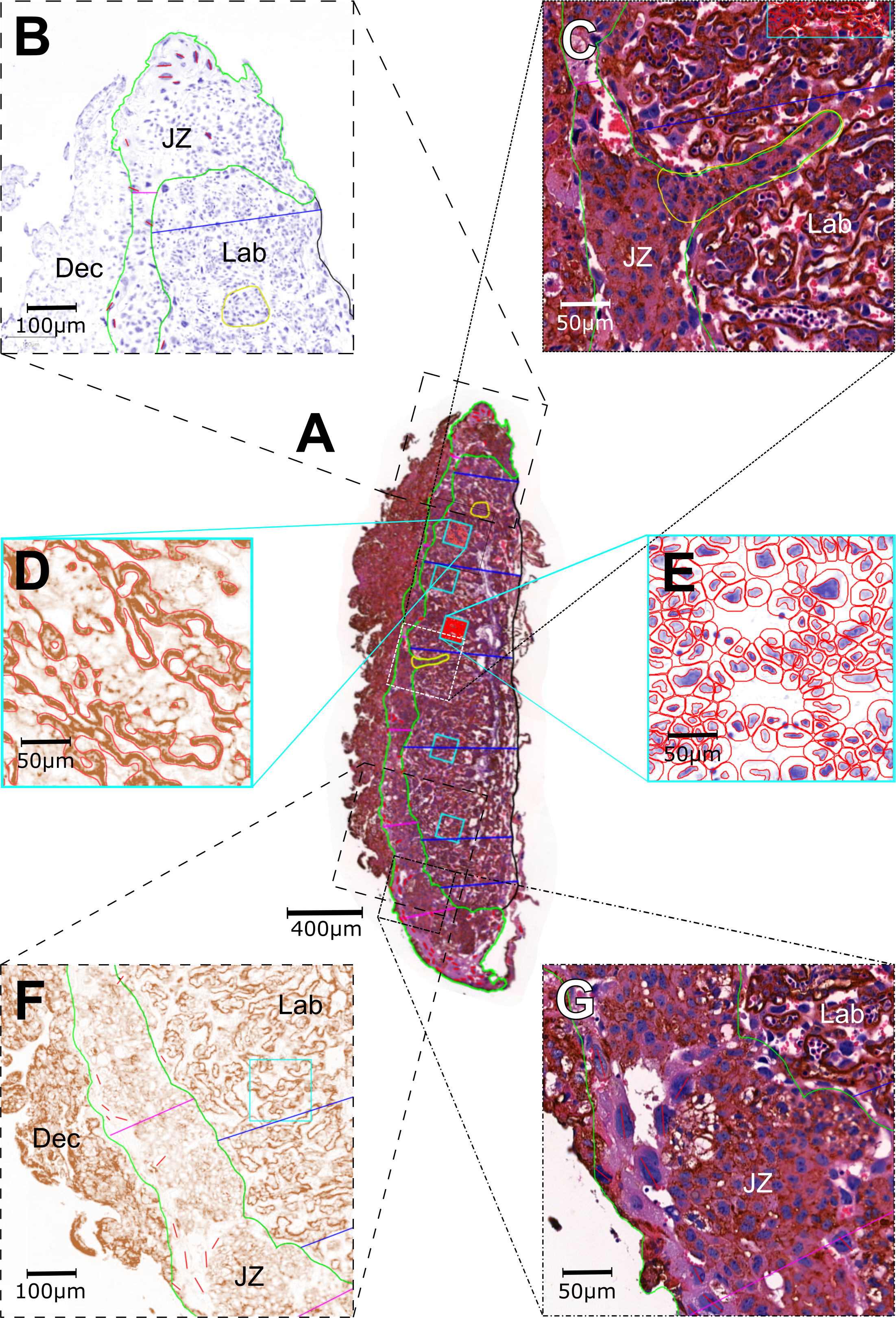

Supplement: S1 Fig — Labyrinth (Lab), junctional zone (JZ), and decidua (Dec) placenta areas are indicated. Annotations throughout indicate junctional zone area (green), junctional zone thickness (pink lines) labyrinth area (black), labyrinth thickness (blue lines) spongiotrophoblast inclusions (yellow), giant cell nuclei (straight red lines), labyrinth quantification areas (turquoise squares). (A) Whole mouse placenta section stained with IL-B4 and H&E at 1x magnification. (B) Haematoxylin channel at 5x magnification showing junctional zone (pink lines) and labyrinth (blue lines) thickness measurements and contrasting nuclei morphology in the placenta areas. (C) IL-B4 and H&E channels at 10x magnification showing a spongiotrophoblast inclusion (yellow). (D) DAB channel at 10x magnification showing labyrinth basal membrane quantification (red enclosures). (E) Haematoxylin channel at 10x magnification showing labyrinth cell quantification (red circles). (F) DAB channel at 5x magnification showing contrasting IL-B4+ tissue morphology in the placenta areas. (G) IL-B4 and H&E channels at 10x magnification showing giant cells (straight red lines). (TIF) [file pone.0233007.s001.tif]

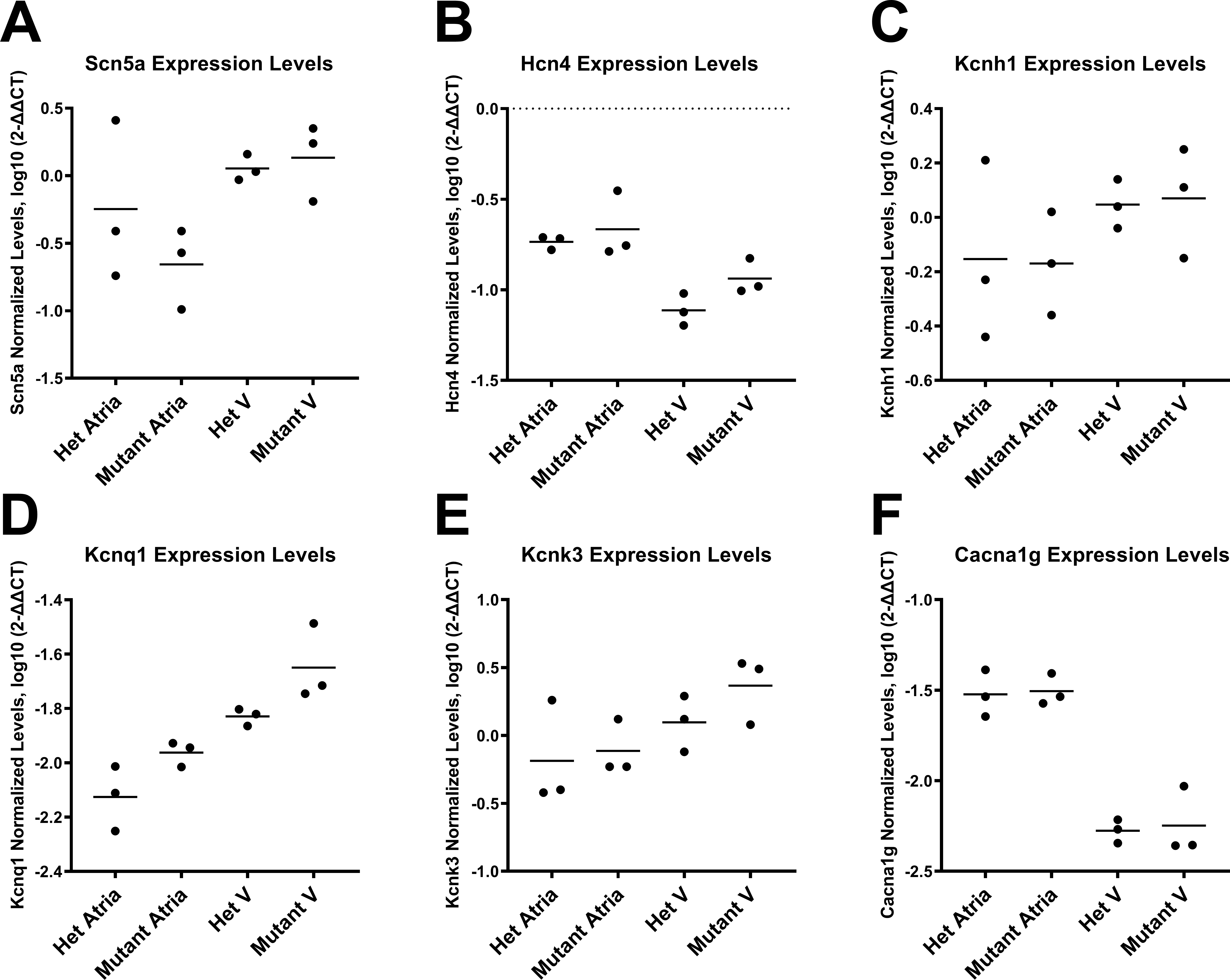

Supplement: S2 Fig — Relative mRNA expression of (A) Scn5a (A), (B) Hcn4 (B), (C) Kcnh1 (C), (D) Kcnq1 (D), (E) Kcnk3 (E), or (F) Cacna1g (F) in heterozygous and mutant hearts presented as log transformed values log10 (2-ΔΔCT). (Het—Heterozygous, V—Ventricles). (TIF) [file pone.0233007.s002.tif]

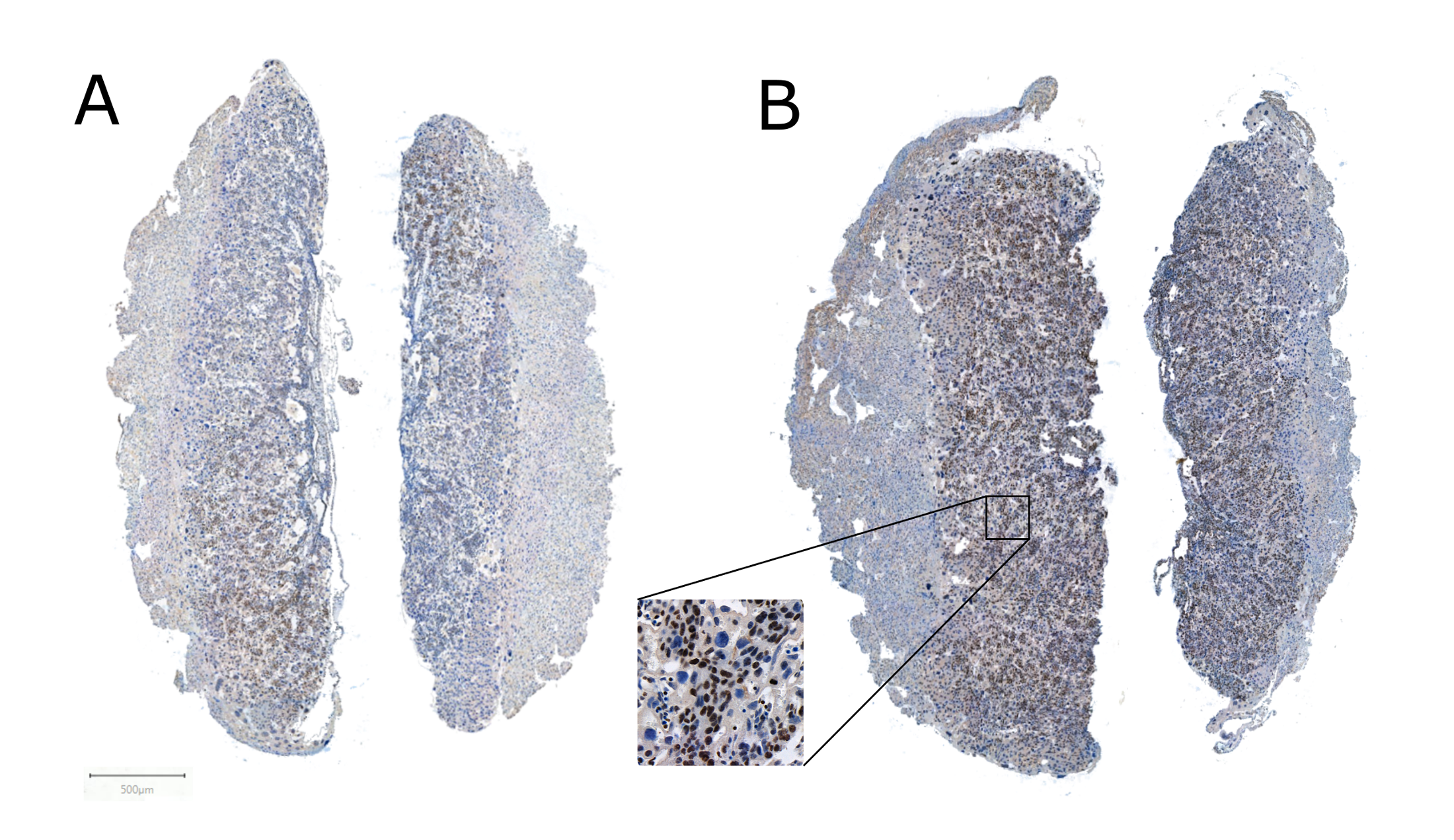

Supplement: S3 Fig — Example images of the tissue sections of (A) heterozygous and (B) homozygous mutant mouse placentas captured at x1 magnification. Arrows indicate Ki67 positive cells. Scale bar = 500μm. (TIF) [file pone.0233007.s003.tif]

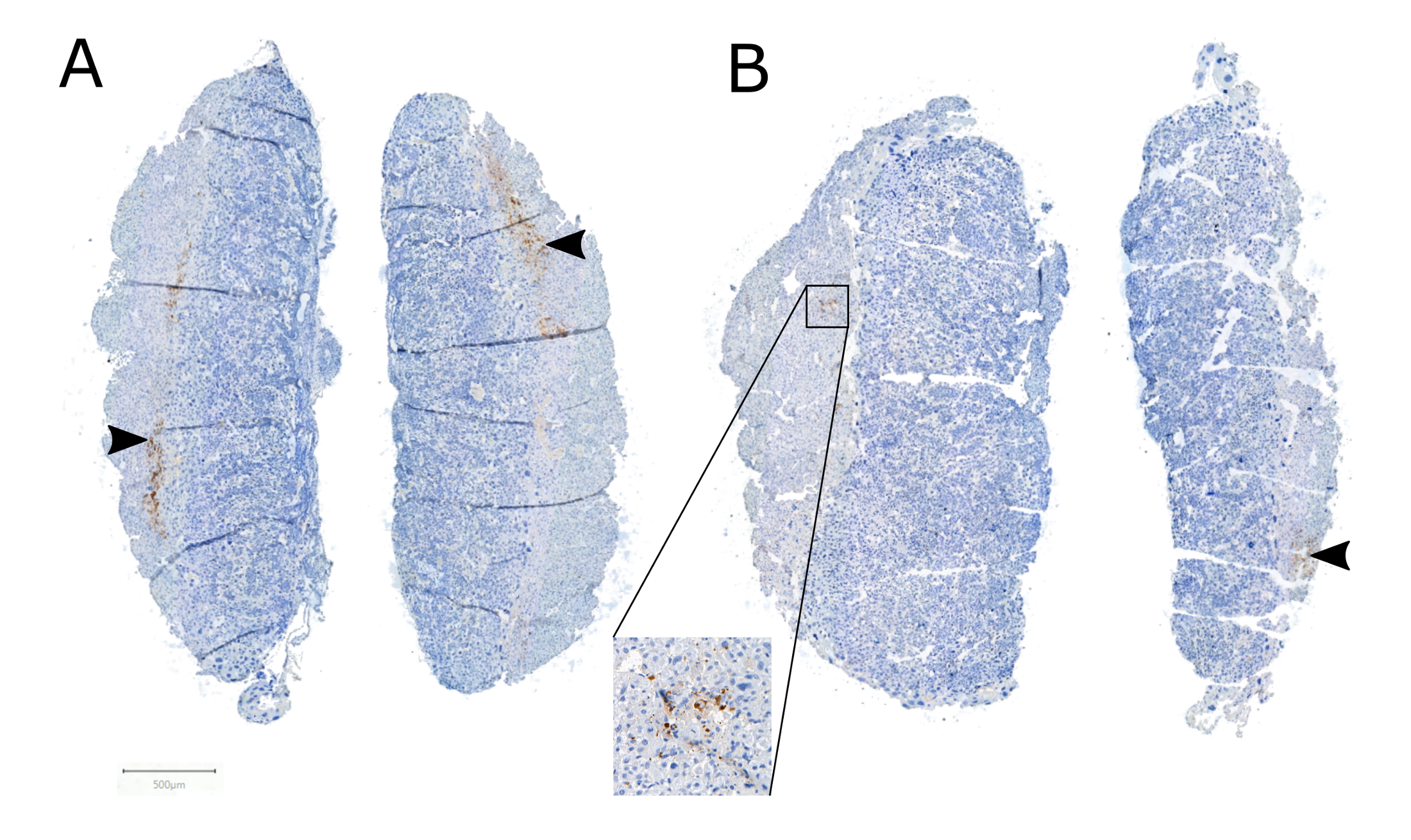

Supplement: S4 Fig — Example images of the tissue sections of (A) heterozygous and (B) homozygous mutant mouse placentas captured at x1 magnification. Arrows indicate Cas3 positive cells. Scale bar = 500μm. (TIF) [file pone.0233007.s004.tif]

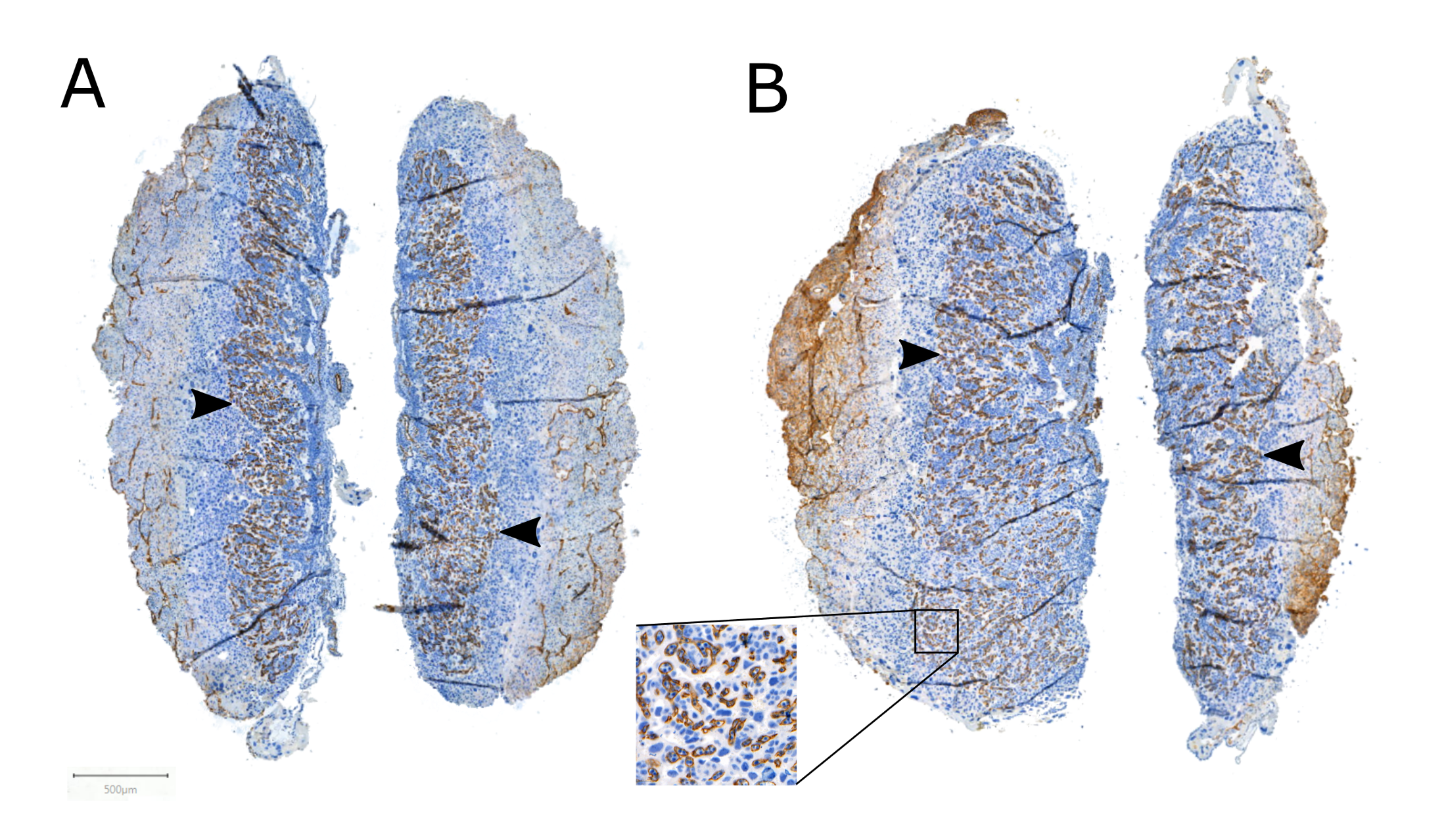

Supplement: S5 Fig — Example images of the tissue sections of (A) heterozygous and (B) homozygous mutant mouse placentas captured at x1 magnification. Arrows indicate vessels. Scale bar = 500μm. (TIF) [file pone.0233007.s005.tif]
